# Prise en charge psychologique d'un cas du syndrome de Prader Willi: cas d'une jeune fille marocaine

**DOI:** 10.11604/pamj.2019.33.115.13726

**Published:** 2019-06-14

**Authors:** Lamyaa Benchikhi, Hind Nafiaa, Abdelaaziz Zaroual, Abderrazzak Ouanass

**Affiliations:** 1 Centre des Etudes Doctorales des Sciences de la Vie et de la Santé, Faculté de Médecine et de Pharmacie de Rabat, Université Mohammed V, Rabat, Maroc

**Keywords:** Prader Willi, obésité, psychothérapie, Prader Willi, obesity, psychotherapy

## Abstract

Le syndrome de Prader Willi est une maladie génétique rare qui se manifeste par l'apparition d'une hyperphagie avec un risque d'obésité morbide, des difficultés d'apprentissage et des troubles du comportement, voire des troubles psychiatriques majeurs. L'objectif de cette analyse de cas est de faire une revue de littérature à propos du syndrome de Prader Willi, ainsi que de mettre en exergue ses conséquences sur la vie quotidienne, tant sur le plan physique que psychiatrique et ses moyens de prise en charge. On rapporte ici le cas d'une jeune fille marocaine, âgée de 11ans, qui présente le syndrome de Prader Willi, qu'on a accompagnée durant sa vie quotidienne, bénéficiant d'une prise en charge biopsychosociale globale, lui permettant ainsi une meilleure qualité de vie. Nous soulignons ici le rôle primordial et central que joue le psychothérapeute dans la prise en charge du syndrome de Prader Willi, à travers l'accompagnement psychologique, les entretiens motivationnels et les techniques de la thérapie cognitive et comportementale. Le syndrome de Prader Willi est une maladie à manifestations psychiatriques importantes, qui doivent être prises en charge par une équipe multidisciplinaire dont fait partie le psychologue ou le psychiatre.

## Abstract (en)

Prader Willi syndrome is a rare genetic disease. Symptoms include binge eating disorder associated with the risk of morbid obesity, learning difficulties and behavioral problems or even major psychiatric disorders. The purpose of this case study is to conduct a literature review on Prader Willi syndrome and to highlight its impact on daily life, both on physical and mental health, as well as its management. We report the case of an 11-year old Moroccan girl presenting with Prader Willi syndrome. She was assisted in performing activities of daily living by using a comprehensive biopsychosocial approach which improved her quality of life. We here emphasize the primary and central role of the psychotherapist in the treatment of patients with Prader Willi syndrome which is based on counselling, motivational interviewing and cognitive behaviour therapy techniques. Prader Willi syndrome is a disease with severe psychiatric symptoms. Patient’s care requires multidisciplinary team approach involving psychologist or psychiatrist.

## Introduction

Le surpoids et l'obésité se définissent comme étant une accumulation anormale ou excessive de graisse corporelle qui représente un risque pour la santé. L'indice de masse corporelle (IMC) est un moyen simple de mesurer l'obésité dans la population: il correspond au poids de la personne (en kilogrammes) divisé par le carré de sa taille (en mètres). Une personne dont l'IMC est supérieur ou égal à 25 est considérée comme étant en surpoids. Une personne ayant un IMC de 30 ou plus est généralement considérée comme obèse. Le surpoids et l'obésité sont des facteurs de risque majeurs pour un certain nombre de maladies chroniques, parmi lesquelles le diabète, les maladies cardio-vasculaires et le cancer. Selon l'Organisation Mondiale de la Santé, entre 1980 et 2014, l'incidence de l'obésité s'est multipliée par deux et le surpoids a touché 1,9 milliards de personnes de 18 ans et plus. En 2014, 41 millions d'enfants de moins de 5 ans étaient en surpoids ou obèses [1]. La maladie de Prader Willi est une maladie rare, selon les auteurs, l'estimation de sa fréquence est d'environ 1 naissance sur 15.000 à 20.000 [2]. Ce sont les médecins suisses A. Prader, H. Willi et A. Labhart qui sont les premiers à décrire le syndrome de Prader Willi, en 1956. Pendant longtemps, le diagnostic reposait uniquement sur la présence d'une série de critères cliniques. Ce n'est qu'en 1981 que le docteur Ledbetter a pu isoler une délétion du chromosome 15 chez ces patients, puis en 1989, que des analyses moléculaires ont révélés qu'elle concernait celui d'origine paternelle. Enfin, depuis 1989, une disomie maternelle de ce même chromosome est reconnue comme seconde forme génétique possible du syndrome.

Dans cette maladie, l'obésité a un profil particulier car elle s'associe une hypotonie néonatale, obésité sévère précoce, un hypogonadisme, un retard statural ou à un infléchissement de la vitesse de croissance, des troubles du comportement et un profil psychologique particulier rendant difficile la prise en charge pour le patient, son entourage de même que celle de l'équipe soignante. D'autre part, elle se constitue dès la deuxième année de vie et évolue de façon très rapide. Par ailleurs, la masse maigre est faible et la répartition des graisses est singulière avec une disposition au niveau du visage, de l'abdomen, des hanches, des fesses et des cuisses [3]. Une prise de poids excessive et une tendance à beaucoup trop manger apparaît progressivement, mais à un rythme différent d'un enfant à l'autre. Cependant, le poids a tendance à augmenter sans que l'appétit augmente. Vers 4 à 5 ans, l'appétit et donc l'absorption de calories augmentent. C'est en général plus tard, vers 8 ans, que l'enfant perd la sensation d'être rassasié et se met à manger beaucoup trop (hyperphagie). En même temps, son organisme a besoin de moins de calories que les autres pour fonctionner: ses besoins caloriques sont inférieurs à la moyenne (d'un tiers environ). Tout ceci contribue à une prise de poids rapide et, en l'absence de régime alimentaire strict, l'enfant a tendance au surpoids, voire à l'obésité. Il n'existe pas actuellement de traitement capable de guérir ce syndrome. Cependant, dès que le diagnostic est établi, la prise en charge précoce et par différents professionnels travaillant en concertation améliore l'évolution de la maladie et diminue les complications. La prise en charge doit être mise en place dès que le diagnostic est confirmé. Elle fait intervenir une équipe médicale, composée de différents spécialistes, entre autres, pédiatre, pédopsychiatre, psychologue, gastroentérologue, endocrinologue, neurologue. Nous allons nous pencher dans notre article sur un cas clinique particulièrement intéressant du syndrome de Prader Willi.

## Patient et observation

Il s'agit donc d'une jeune fille âgée de onze ans, non scolarisée. Issue d'un niveau socio-économique moyen, de père commerçant et mère femme au foyer. Elle est la troisième d'une fratrie de quatre dont un garçon et deux filles. A l'initiative de quelques bienfaiteurs, la fille a été admise dans une clinique privée à Rabat pour obésité morbide chiffrée à un poids de 155kg, avec un indice de masse corporelle à 63,69kg/m^2^, affectant sa motricité et son insertion scolaire et sociale. Le début des symptômes remonterait à l'âge de quatre ans, alors que la fille pesait 46kg dus essentiellement à une hyperphagie et des grignotages permanents. De plus, la patiente est connue par ses gestes répétitifs de grattage des parties de son corps à chaque frustration ou pendant ses crises d'angoisse. Parallèlement, elle présente une obsession idéative qui tourne autour de la nourriture avec des gestes quotidiens de faire et défaire des nœuds par des foulards auxquels elle s'attache le long de la journée. Dès le début des symptômes, la patiente a fait le tour des médecins et le diagnostic du syndrome de Prader Willi a été retenu après avoir retracé l'histoire de sa maladie. Jusqu'à l'âge de six ans, la patiente n'a pas été prise en charge sur le plan diagnostique et thérapeutique et par conséquent, elle n'a pas cessé de prendre davantage du poids et d'une façon disproportionnée avec persistance des symptômes associés, surtout avec une attitude permissive et de soumission de la mère aux troubles de conduites alimentaires de sa fille.

Cette situation s'est répercutée négativement sur sa scolarité qui a débuté tardivement vers l'âge de sept ans dans une classe d'inclusion scolaire où elle n'a pu restée que deux ans. La petite fille n'a pas accepté de se retrouver avec des enfants en situation d'handicap et subir en plus des maltraitances quotidiennes de la part de ses camarades de classe. En outre, la patiente se serait vue obligée de réduire son activité physique à cause de son surpoids, en restant parfois alitée à son domicile, un appartement situé au quatrième étage dans un immeuble sans ascenseur; chose qui l'oblige de rester prisonnière chez elle, hormis quelques sorties hebdomadaires après avoir passé une heure pour monter les escaliers, avec des pauses fréquentes et prolongées. Désirant venir en aide à sa fille, son père n'a pas hésité à contacter la presse. Grâce à la médiatisation de son cas, la patiente a été prise en charge par une équipe de professionnels bénévoles dans une clinique à Rabat. Tenant compte de son âge et de la contre-indication de la chirurgie de l'obésité, la patiente a bénéficié d'un séjour thérapeutique incluant; les examens médicaux, le suivi diététique, les séances de massage et de renforcement musculaire et l'accompagnement psychologique que nous lui avons offert. Au cours de son admission à la clinique, la jeune patiente avait reçu la visite des différentes personnalités célèbres. Sur le plan psychologique, une évaluation psychologique a été entreprise au moyen de tests et échelles: échelle de Rosenberg d'estime de soi, échelle de qualité de vie SF12, échelle de l'image de corps. Ensuite, la patiente avait bénéficié d'un accompagnement psychologique comportant; des séances d'hypnose médicale, de relaxation, thérapie cognitivo-comportementale. Grâce au travail bénévole de l'équipe pluridisciplinaire, la patiente a pu perdre 15Kg au terme de deux mois.

Avant d'aborder le processus thérapeutique, la patiente a passé l'échelle d'estime de soi de Rosenberg qui vise à évaluer la valeur que se fait la personne d'elle-même. Aussi, l'intéressée a passé une autre échelle d'image de corps pour mesurer le niveau de comparaison sociale concernant l'apparence physique et les attitudes sociales envers l'apparence physique et en dernier lieu, une échelle de qualité de vie SF12 a été administrée dans le but d'explorer la perception qu' a l'individu sur son existence dans son milieu. Les résultats obtenus dans les différentes échelles ont montré: une faible estime de soi avec un score de 20 pour l'échelle d'estime de soi. Un score très bas pour la comparaison sociale concernant l'apparence physique ainsi que pour les attitudes sociales envers l'apparence physique; et une qualité de vie altérée.

Au terme de cette évaluation psychologique, un protocole thérapeutique a été mis en place et qui est axé sur trois volets principaux: l'accompagnement; le suivi psychologique; séances de relaxation et de reconstruction cognitive inspirée de la thérapie cognitivo- comportementale.

## Discussion

Notre patiente a été soumise au début à des échelles d'évaluation psychologique qui ont confirmé de faibles scores au niveau de l'estime de soi, l'image de corps et la qualité de vie. Au regard de ces résultats, le travail thérapeutique a été centré sur l'accompagnement, la relaxation et des séances de thérapie cognitivo-comportementale. Après une prise en charge pluridisciplinaire, la jeune fille a perdu 15Kg de son poids avec estompement des symptômes observés au début de son admission à la clinique. Néanmoins, quelques mois après sa sortie, la patiente a rechuté avec réapparition plus intense de signes. Sachant très pertinemment qu'elle n'a bénéficié d'aucun suivi en ambulatoire. Ce qui montre que cette maladie nécessite un accompagnement en particulier psychologique.

### Processus thérapeutique

#### L'accompagnement psychologique

Afin d'instaurer une alliance thérapeutique, la première rencontre avec la patiente s'est fait en présence de son médecin traitant après son acceptation. Elle a exprimé son souhait de devenir belle et c'est la raison de son admission à la clinique. Lors de mes premières rencontres, j'ai observé une angoisse envahissante et une difficulté de verbaliser son malaise qu'à travers le dessin où elle a dessiné une belle fille mince en précisant que la fille sur le dessin n'est pas elle. Aussi, elle a refusé de dessiner son corps. Sachant bien que le dessin est une sorte d'évocation répétitive des représentations et des images inconscientes. Après cette première démarche, il s'illustre au premier plan une désorganisation somatique et un passage à l'acte comportemental. La situation de la patiente paraît bloquée réduisant le travail psychique à un fonctionnement quasi opératoire. Ce sont des faits extérieurs: des événements de la réalité, qui marquent sa vie et s'imposent du dehors entraînant chez elle des réactions en quelque sorte mécaniques face auxquelles elle ne peut rien. Au fil des rencontres, la jeune fille est arrivée à communiquer et verbaliser ses vécus et ses ressentis. Mais, elle reste toujours vulnérable sur le plan relationnel et social. L'hypersensibilité marque les réactions de la jeune fille que engendre une souffrance psychique surtout avec la comparaison aux autres sur le plan physique, stigmatisée par le poids du regard de l'autre [4, 5]. Généralement, les cas de syndrome de Prader Willi présentent un manque d'empathie et des difficultés à nouer des liens au sein de leur entourage [6]. En outre, notre patiente a montré un attachement morbide envers des personnes particulières avec des demandes affectives massives et où elle exprime ses émotions d'une façon exagérée et considère ses relations plus intimes qu'elles ne le sont en réalité. Thuilleaux évoque à ce propos une «hypertrophie de la vie affective» [7]. Face à sa fragilité relationnelle et sociale, la patiente a été intégrée au sein d'un groupe de parole composé d'enfants admis à la clinique pour différentes maladies. L'évolution de la jeune fille a été significative dans la construction de son discours et son interaction au sein du groupe. Sachant pertinemment que le contrôle de désir alimentaire est difficile et devant la contre indication des médicaments coupe-faim dans ce cas, notre intervention s'est limitée à la psycho-éducation en visant les parents qui doivent suivre le même régime alimentaire que la patiente avec des horaires bien précis pour atténuer l'effet de la frustration face à ces restrictions alimentaires [8]. Cette démarche est prise en tenant en considération que les parents restent impuissants devant l'augmentation de l'appétit puisque les idées obsessionnelles à propos de la nourriture poussent à sa recherche permanente. Ces préoccupations alimentaires diffèrent dans la fréquence et l'intensité suivant les personnes [9]. Sous crainte d'une déception post hospitalisation due essentiellement à la diminution de la fréquence des visites des personnes intéressantes, le médecin psychiatre a décidé d'interdire les visites. Cette mesure a déclenché un état d'agitation chez la patiente avec des grattages jusqu'au saignement au niveau des bras, jambes et sein gauche. Ce comportement pourrait être le signe d'un acte d'automutilation comme une conséquence à l'insensibilité à la douleur mais dans notre cas, l'hypothèse d'un lien étroit entre le grattage et l'anxiété pourrait être la plus probable car il se déclenche devant chaque frustration ou contrariété [10]. Notre action face aux réactions de la patiente s'est basée essentiellement sur les techniques comportementales et de relaxation, un apprentissage de contrôle des émotions et de la gestion des situations de crises.

Les séances de thérapies cognitivo comportementales avaient pour but le contrôle des stimuli qui vise: 1) à mieux maîtriser le comportement alimentaire et à mieux gérer les aliments à disposition; 2) Etablissement du carnet alimentaire (apports, rythme, pensées, émotions); 3) Restructuration cognitive; 4) Reformulation des pensées dysfonctionnelles concernant le poids, l'alimentation, l'image du corps et l'estime de soi. Au terme de son séjour dans la clinique et grâce au travail pluridisciplinaire, la patiente a pu perdre 15kg de son poids. Certes, cet objectif associé à d'autres reflètent la qualité de la prestation de l'ensemble des acteurs impliqués dans la prise en charge, néanmoins, le travail post-hospitalisation demeure d'une importance capitale dans la mesure où il permettra de maintenir les résultats réalisés d'où une démarche psychologique s'impose à la patiente comme à son entourage. Ce protocole comporte une sensibilisation sur l'importance du maintien de nouveaux réajustements apportés aux conduites alimentaires. La pratique des techniques de relaxation à domicile pour un meilleur contrôle de soi sans omettre pour autant la mise en place d'un planning pour les activités quotidiennes facilitant ainsi une intégration sociale et un épanouissement personnel.

## Conclusion

En définitive, la prise en charge du syndrome de Prader Willi se limite à atténuer les retentissements psychologiques, somatiques et sociaux de ce syndrome et améliorer les conditions de vie des patients et celles de leurs familles. Cette prise en charge doit être précoce, multidisciplinaire et reposer essentiellement sur l'accompagnement et la prévention de nombreuses complications [11]. Nous avons pu constater que les symptômes observés sur le sujet du syndrome de Prader Willi sont complexes et nombreux. A travers l'ensemble des travaux réalisés au sujet du syndrome de Prader Willi, l'intérêt apporté par la communauté scientifique internationale reste en croissante évolution. Cependant, il reste beaucoup à faire pour donner des explications à la maladie et élucider les différents mécanismes qui interviennent dans son évolution.

## Conflits d’intérêts

Les auteurs ne déclarent aucun conflit d'intérêts.
